# The Prevalence and Determinants of Hepatic Steatosis Assessed by Controlled Attenuation Parameter in Thai Chronic Hepatitis C Patients

**DOI:** 10.1155/2020/8814135

**Published:** 2020-11-02

**Authors:** A. Sirinawasatien, T. Techasirioangkun

**Affiliations:** Division of Gastroenterology, Department of Medicine, Rajavithi Hospital, College of Medicine, Rangsit University, Bangkok, Thailand

## Abstract

**Aims:**

To describe the prevalence of hepatic steatosis using a controlled attenuation parameter (CAP) and to identify the determinants associated with steatosis in Thai chronic hepatitis C patients. *Patients and Methods*. An observational study was conducted among consecutive chronic hepatitis C patients who underwent vibration-controlled transient elastography (VCTE, FibroScan®) with CAP and followed up at Rajavithi Hospital, Bangkok, Thailand, between June 2018 and May 2019. Hepatic steatosis (i.e., steatosis grades S1-3) was defined by the CAP cutoff value of ≥248 (dB/m). VCTE with CAP assessments and medical records were retrospectively reviewed, and the prevalence and determinants of hepatic steatosis were analyzed.

**Results:**

A total of 197 eligible patients, of whom 127 (64.5%) were male, were included. The mean age was 54.52 years (SD 9.49 years), and 41.1% of subjects had a body mass index ≥ 25. The prevalence of hepatic steatosis was 26.9%. The mean liver stiffness measurement (LSM) was 21.50 kPa (SD 15.58 kPa), and 61.9% of the study population had cirrhosis, which was defined as LSM ≥ 12.5 kPa. Genotype (GT) 3 was predominant at 40.1%, followed by GT1 at 38.1% and GT6 at 21.8%. The median serum hepatitis C virus viral load was 1,100,000 IU/mL (range 5,824-20,436,840). The significant determinants of hepatic steatosis were obesity (aOR 8.58 (95% CI: 3.41-21.54)) and diabetes mellitus (aOR 3.30 (95% CI: 1.24-8.78)).

**Conclusion:**

A large proportion of these Thai chronic hepatitis C patients (26.9%) had hepatic steatosis, which was strongly associated with host metabolic factors, e.g., obesity (BMI ≥ 25) and diabetes mellitus. These cofactors contributed to the progression of liver disease to cirrhosis and required concurrent management with antiviral therapy.

## 1. Introduction

The hepatitis C virus (HCV) has been found to be a major cause of chronic liver disease, cirrhosis, and hepatocellular carcinoma with the worldwide population infected with HCV estimated at 71 million individuals [1]. Nonalcoholic fatty liver disease (NAFLD) is increasingly recognized as the hepatic manifestation of metabolic syndrome [2], characterized by hepatic steatosis, which encompasses a wide spectrum of diseases ranging from simple steatosis to steatohepatitis [3]. The prevalence of steatosis in HCV-infected patients has been reported to be approximately 50% [4], much higher than that of the general population (20-30%) [5, 6]. The underlying mechanism of steatosis occurrence is not entirely understood, but in the HCV genotype (GT) 1, it acts via inducing insulin resistance [7], while in the HCV GT3, it is predominantly a direct virus-related effect [8]. This concomitant condition in HCV patients can accelerate liver fibrosis progression [9], and it is also associated with a lower virological response to antiviral therapy [10].

A liver biopsy remains the gold standard for the assessment of hepatic steatosis and other liver diseases; however, it has several disadvantages, including its invasiveness, its associations with some rare serious complications, and its susceptibility to sampling variability [11, 12]. A controlled attenuation parameter (CAP), based on the properties of ultrasound signals acquired by vibration-controlled transient elastography (VCTE) (FibroScan®, Echosens, Paris), is a novel technique that measures the attenuation, caused by fat, of ultrasound propagation in the liver [13]. The results from previous studies demonstrated that CAP performs well in the detection and quantification of steatosis in many types of chronic liver disease [14, 15]. Moreover, VCTE provides an assessment of CAP together with liver stiffness measurement (LSM), which has recently been increasingly used for assessing liver fibrosis in HCV-infected patients with a >90% negative predictive value for ruling out cirrhosis [16], as well as the noninvasive score; fibrosis-4 (FIB-4) index for liver fibrosis is a useful diagnostic tool in the estimation of advanced fibrosis; therefore, it is recommended to use FIB-4 and VCTE in the management of patients infected with HCV [17].

Thus far, there have been few studies of the extent of hepatic steatosis in HCV-infected patients in Thailand, and the present research is aimed at describing its prevalence in Thai chronic viral hepatitis C patients using a noninvasive test (CAP) and at identifying the determinants associated with it.

## 2. Materials and Methods

### 2.1. Study Design

This observational study was conducted by reviewing the medical records of all chronic hepatitis C patients who underwent VCTE with CAP and followed up at the liver clinics of the Department of Medicine at Rajavithi Hospital, a tertiary referral center in Bangkok, Thailand, between June 2018 and May 2019. All HCV-infected patients who have persistent viremia defined by the detection of HCV RNA in plasma and had not received previous HCV treatment were indicated to undergo VCTE with a CAP in the present study. The study was carried out in accordance with the ethical principles of the Declaration of Helsinki and was reviewed and approved by the ethics committee of Rajavithi Hospital (No. 087/2563).

### 2.2. Participants

The inclusion criteria were patients who (i) were aged older than 18 years, (ii) were diagnosed as having HCV infection by detection of HCV RNA in plasma, and (iii) had not received previous HCV treatment before VCTE was performed. We excluded patients who (i) had hepatitis B virus (HBV) or human immunodeficiency virus (HIV) coinfection; (ii) had a history of alcohol abuse or abstinence from alcohol for less than 6 months; (iii) had a positive test for antinuclear antibody, antismooth muscle antibody, or antimitochondrial antibody; (iv) had a significant elevation of liver enzymes (aminotransferases > 5-fold the upper limit of normal (ULN)); or (v) had an invalid VCTE result.

### 2.3. Liver Stiffness Measurement (LSM) and Controlled Attenuation Parameter (CAP)

All patients in the present study had VCTE and CAP performed by a single operator (Thanaya Techasirioangkun, RN) who had experience of more than 1000 cases using a FibroScan® device (Echosens, France). This device sends a shear wave through the liver, after which the propagation of this wave is assessed, as its velocity is directly related to tissue stiffness because higher liver stiffness causes the shear wave to move faster. After fasting for at least 2 hours, all participants had LSM with the standard adult M-probe VCTE placed perpendicular to the skin through the 9^th^-11^th^ intercostal space on the liver's right lobe while the patient remained in the supine position with his/her right arm maximally abducted. LSM was expressed in kilopascal (kPa) with a range from 2.5 to 75 kPa. An accepted VCTE result requires at least 10 validated measurements, a success rate (the ratio of valid measurements to the total number of measurements) above 60%, and an interquartile range (IQR, which reflects variations among measurements) of less than 30% of the median value [18]. The stages of liver fibrosis were categorized into histopathological staging by the METAVIR scoring system, with minimal fibrosis (F0-1), significant fibrosis (*F* ≥ 2), advanced fibrosis (*F* ≥ 3), and cirrhosis (*F* = 4) using LSM cutoffs of <7.0 kPa, 7.0-10.9 kPa, 11.0-12.4 kPa, and ≥12.5 kPa, respectively [19–21].

CAP for the assessment of liver steatosis is based on the properties of ultrasound signals acquired by VCTE, as fat affects ultrasound propagation so that the degree of ultrasound attenuation correlates with hepatic fat infiltration [13, 15]. Using the same radiofrequency data and the same region of interest used to assess liver stiffness, CAP values were expressed in decibels per meter (dB/m) (range 100-400 dB/m) [13] and were interpreted only when the associated LSM value was valid. In accordance with a recent meta-analysis of studies containing histology verified CAP data for grading of steatosis (S0-S3), the CAP cutoff value of ≥248 (dB/m) was used for distinguishing steatosis grade more than S0, which was defined by the number of affected hepatocytes: S0 (<5 or 10%), S1 (5 or 10-33%), S2 (34-66%), and S3 (>66%) [22].

### 2.4. Clinical Evaluation and Laboratory Data

Data from patients who underwent both VCTE and CAP within 4 weeks were collected, retrospectively, from medical records for description as well as a comparison between CAP-classified groups of patients with and without hepatic steatosis (i.e., S1-3 versus S0). Demographic and baseline characteristics (e.g., age, gender, body mass index (BMI), comorbid diseases, and LSM) and laboratory data (e.g., serum albumin (g/dL), total bilirubin (mg/dL), aspartate transaminase (AST) level (U/L), alanine transaminase (ALT) level (U/L), alkaline phosphatase (ALP) level (U/L), serum creatinine (mg/dL), hemoglobin (g/dL), white blood cell count (WBC) (/mm^3^), platelet count (×10^9^/L), international normalized ratio (INR), HCV RNA level (IU/mL), and HCV-genotype) were included. The upper limit of normal (ULN) for AST, ALT, and ALP was defined as 40 U/L, 40 U/L, and 129 U/L, respectively. BMI of <23, 23-24.9, and ≥25 kg/m^2^ were categorized as normal, overweight, and obese, respectively [23]. The serum HCV viral load was measured using real-time HCV assays by polymerase chain reaction using COBAS® AmpliPrep/COBAS® TaqMan® HCV Test (Roche Molecular Diagnostics, CA, USA), with the range from 15 to 1.7 × 10^8^ IU/mL. HCV genotype was analyzed by direct sequencing of the HCV core gene using the ABI3500 Genetic Analyzer instrument (Applied Biosystem, CA, USA).

The FIB-4 score combines biochemical values (AST, ALT, and platelet count) and age, which was initially found the ability to exclude advanced fibrosis among patients with hepatitis C [24]. Moreover, FIB-4 is recommended for the first line triaging of those patients considering its easy calculation in both patients with elevated or normal transaminases [25, 26]. The FIB-4 score was calculated as previously described [27]; those patients who scored <1.3 and >2.67 on the FIB-4 were considered at low and high risks of advanced fibrosis, respectively [27].

In terms of comorbidity, hypertension was defined as a systolic blood pressure ≥ 140 mmHg or diastolic blood pressure ≥ 90 mmHg, diabetes mellitus was defined as fasting plasma glucose ≥ 126 mg/dL or glycated hemoglobin ≥ 6.5%, and dyslipidemia was defined as an elevation of serum total cholesterol > 200 mg/dL or triglyceride > 150 mg/dL or low-density lipoprotein cholesterol > 100 mg/dL.

### 2.5. Statistical Analysis

All statistical analyses were performed using SPSS software version 17.0 (SPSS Inc., Chicago, IL). Demographic data and baseline characteristics were analyzed using descriptive statistics, and categorical data were assessed by the chi-squared test or Fisher's exact test as appropriate, while continuous data were analyzed by the independent *t*-test or the Mann-Whitney *U* test. The determinants associated with hepatic steatosis in chronic hepatitis C patients analyzed by multivariable logistic regression analysis were BMI (reference, <23 kg/m^2^), diabetes mellitus, AST (reference, ≤1 × ULN), ALP (reference, ≤1 × ULN), and FIB-4 (reference, <1.3), and results were presented as adjusted odds ratios (aOR) with 95% confidence intervals. All statistical examinations were two-tailed with a *P* value < 0.05 considered statistically significant.

## 3. Results

Two hundred chronic hepatitis C monoinfection patients were identified and assessed for eligibility. Three patients (1.5%) with invalid VCTE results were excluded, and the remaining 197 patients who had not received any antiviral therapy and had complete laboratory data were included in the study. Patients with hepatic steatosis defined by the CAP value of ≥248 dB/m accounted for 26.9% of the study population.

The mean liver stiffness was 21.50 kPa (SD 15.58 kPa) with more than half (61.9%) of the study population having cirrhosis defined as LSM ≥ 12.5 kPa. Males accounted for 64.5% of the patients, whose mean age was 54.52 years (SD 9.49). The mean BMI was 24.66 (SD 4.28) while 41.1% of subjects were obese (BMI ≥ 25 kg/m^2^). Regarding comorbid disease, hypertension, diabetes mellitus, and dyslipidemia accounted for 19.8, 13.2, and 8.6% of patients, respectively. In terms of genotype distribution, GT3 was the most common at 40.1%, followed by GT1 at 38.1% and GT6 at 21.8%, and high baseline viral load, defined as HCV RNA ≥ 6,000,000 IU/mL, was found in 11.7% of the patients.

Patients with steatosis had significantly higher BMI than those without it (27.51 (SD 4.21) versus 23.61 (SD 3.82) kg/m^2^, *P* < 0.001), and a significantly higher proportion of obese cases (BMI ≥ 25 kg/m^2^) were noted in the former group than in the latter one (75.5 versus 28.5%, *P* < 0.001). Diabetes mellitus was encountered significantly less frequently in patients without steatosis than in those with it (9.0 versus 24.5%, *P* = 0.004), while HCV genotype and serum HCV viral load were not significantly different between the two groups. Laboratory data showed significantly higher levels of AST, ALP, and FIB-4 in the patients without steatosis than in those with it (*P* < 0.05). LSM and CAP results, together with baseline characteristics and laboratory data of all patients and patients with and without hepatic steatosis, are summarized in Table 1.

Univariate analysis demonstrated that the determinants for hepatic steatosis were obesity and comorbidity with diabetes mellitus, while showing an inverse association with high AST and ALP levels (>1 × ULN), as well as high FIB-4 index (≥1.3). However, multivariable logistic regression analysis showed that only obesity and diabetes mellitus were significant determinants of hepatic steatosis with aOR of 8.58 (95% CI: 3.41-21.54) and 3.30 (95% CI: 1.24-8.78), respectively (Table 2).

## 4. Discussion

The present study included consecutive HCV-infected patients undergoing CAP and identified hepatic steatosis in 26.9% of subjects, which is a lot lower than that reported (about 50%) in other studies [4, 9]. Furthermore, we demonstrated that in our HCV patients, steatosis was strongly associated with obesity and diabetes mellitus. Several studies have noticed a significant correlation between HCV genotype 3 infection and the presence of steatosis [9, 28, 29], the severity of which may be related to levels of HCV RNA in patients infected with genotype 3 [9]; however, our study did not confirm this relationship.

The pathogenesis of hepatic steatosis in chronic hepatitis C patients is not exactly established even though it is associated with both viral factors (HCV genotype 3) and host metabolic factors. Obesity is associated with resistance to the effects of insulin on peripheral glucose and fatty acid utilization, so-called insulin resistance, and may also lead to type 2 diabetes mellitus and contribute to the development of steatosis [30]. In previous studies, a high BMI was reported to be one of the factors associated with hepatic steatosis [9, 28, 29], and we found similar results with multivariate analysis in the present study, in which obesity (BMI ≥ 25) was a significant determinant of hepatic steatosis with an aOR of 8.58 (95% CI: 3.41-21.54). Ortiz et al. studied the annual change in the fibrosis stage (fibrosis progression rate) on liver histology in chronic hepatitis C patients, and they discovered that obesity (BMI ≥ 25) was the factor that predicted rapid disease progression [31]. In view of the current pandemic of obesity, these data emphasize that weight reduction may play a significant role in hepatitis C management.

Diabetes mellitus is common in chronic hepatitis C patients, and a recent meta-analysis to determine the prevalence of extrahepatic manifestations in patients with HCV infection revealed its most common manifestation to be diabetes mellitus (found in 15% of patients) [32]. Similarly, in a nationwide population-based register study conducted in Sweden among patients with chronic hepatitis C, the prevalence of diabetes mellitus was almost double that reported in the general population (10.6 versus 5.5%, *P* < 0.05) [33], while our data revealed a prevalence of 13.2%. Several experimental and clinical studies have provided evidence that chronic hepatitis C infection can contribute to and exacerbate insulin resistance through multiple pathogenic mechanisms, including hepatic steatosis, impairment of the insulin signaling pathway, and activation of inflammatory pathways; these can aggravate each other in a vicious cycle that can ultimately result in HCV-associated diabetes mellitus [34]. Bearing in mind this link between these metabolic diseases, it is not surprising that the results of multivariate analysis from the present study revealed that diabetes mellitus was associated with hepatic steatosis with an aOR of 3.30 (95% CI: 1.24-8.78).

Data from various studies have disclosed that steatosis is a common histological feature of chronic hepatitis C infection (35-81%), much more than other chronic liver diseases, including chronic hepatitis B (about 25%) [35]. Compared with HCV-non-3 genotypes, infection with genotype 3 is associated with a higher prevalence at 74% versus 48% and more severe steatosis (S3) at 30% versus 6% [35]. Though the exact mechanism remains elusive, it is suggested that HCV genotype 3 itself can directly induce steatosis by the cytopathic effect of high titer of intrahepatic HCV RNA [36], and the grade of steatosis seems to be related to levels of HCV RNA in genotype 3 infection [9]. However, in the present study, HCV genotype 3 was not significantly related to the presence of hepatic steatosis, and this might be explained by the lower prevalence of steatosis in our study compared with the findings of other studies (26.9% versus 50%) [4, 9]. Our hypothesis is that more than half (61.9%) of the study population had cirrhosis (LSM ≥ 12.5 kPa), which reflects a terminal stage of chronic liver disease in which fatty changes are replaced by fibrotic tissue. This is supported by an important observation that many patients with cryptogenic cirrhosis have been misdiagnosed with NAFLD due to the decrease in histological evidence of steatosis as the disease progresses to cirrhosis [37].

While the present study provides helpful information about the prevalence of hepatic steatosis and associated factors in a large number of Thai chronic hepatitis C patients, our findings had several limitations. Firstly, the observational nature of the study design may have impeded its ability to eliminate confounding factors. Furthermore, as this study examined patients already referred to a tertiary care center, referral bias was always a possibility, in so far as patients with a normal liver biochemical test or vice versa, frank decompensated cirrhosis are less likely to be referred, and these findings may therefore not be generalizable to other patient populations. Finally, lack of histological data was a major limitation of our study, but as all our chronic hepatitis C patients had detectable HCV RNA and were not in an advanced cirrhosis condition, antiviral treatment was justified, and there was no indication for liver biopsy in regular clinical practice.

In conclusion, with the growing epidemic of obesity and NAFLD, our study demonstrated that a significant proportion of Thai chronic hepatitis C patients (26.9%) had hepatic steatosis, which was greatly associated with host metabolic factors, e.g., obesity (BMI ≥ 25) and diabetes mellitus. Therefore, it is vital to raise the awareness of doctors to this important cofactor that contributes to the progression of liver disease to cirrhosis and to offer treatment aimed at metabolic diseases along with antiviral therapy in these patients.

## Figures and Tables

**Table 1:** Baseline characteristics of the patients.

Variables	Patients with hepatic steatosis (*n* = 53)	Patients without hepatic steatosis (*n* = 144)	Total patients (*N* = 197)	*P* value
Age, mean (SD) (y)	52.83 (10.43)	55.15 (9.07)	54.52 (9.49)	0.129
Male sex, *n* (%)	33 (62.3)	94 (65.3)	127 (64.5)	0.695
Liver stiffness, mean (SD) (kPa)	21.47 (18.48)	21.52 (14.44)	21.50 (15.58)	0.985
F0-1, *n* (%)	2 (3.8)	4 (2.7)	6 (3.1)	0.831
F2, *n* (%)	14 (26.4)	43 (29.9)	57 (28.9)	
F3, *n* (%)	4 (7.5)	8 (5.6)	12 (6.1)	
F4, *n* (%)	33 (62.3)	89 (61.8)	122 (61.9)	
BMI				<0.001^∗^
<23, *n* (%)	7 (13.2)	65 (45.1)	72 (36.6)	
23-24.9, *n* (%)	6 (11.3)	38 (26.4)	44 (22.3)	
≥25, *n* (%)	40 (75.5)	41 (28.5)	81 (41.1)	
Mean (SD) (kg/m^2^)	27.51 (4.21)	23.61 (3.82)	24.66 (4.28)	
Comorbid diseases, *n* (%)				
Hypertension	14 (26.4)	25 (17.4)	39 (19.8)	0.157
Diabetes mellitus	13 (24.5)	13 (9.0)	26 (13.2)	0.004^∗^
Dyslipidemia	2 (3.8)	15 (10.4)	17 (8.6)	0.165
HCV genotype, *n* (%)				0.927
1	19 (35.9)	56 (38.9)	75 (38.1)	
3	22 (41.5)	57 (39.6)	79 (40.1)	
6	12 (22.6)	31 (21.5)	43 (21.8)	
HCV RNA, median (range) (IU/mL)	1,594,418 (5,824-10,213,198)	1,021,978 (10,134-20,436,840)	1,100,000 (5,824-20,436,840)	0.810
<6,000,000 IU/mL, *n* (%)	46 (86.8)	128 (88.9)	174 (88.3)	0.684
≥6,000,000 IU/mL, *n* (%)	7 (13.2)	16 (11.1)	23 (11.7)	
Biochemical markers				
Total bilirubin, median (range) (mg/dL)	0.81 (0.11-2.77)	0.73 (0.28-2.76)	0.76 (0.11-2.77)	0.941
AST, median (range) (U/L)	63 (24-164)	80 (23-199)	74 (23-199)	0.009^∗^
ALT, median (range) (U/L)	71 (20-194)	80 (16-195)	77 (16-195)	0.401
ALP, median (range) (U/L)	86 (51-206)	90 (38-243)	89 (38-243)	0.010^∗^
Albumin, median (range) (g/dL)	4.50 (2.30-5.40)	4.30 (2.90-5.50)	4.30 (2.30-5.50)	0.052
INR, median (range)	1.04 (0.90-1.54)	1.08 (0.92-1.39)	1.07 (0.90-1.54)	0.126
WBC, median (range) (/mm^3^)	6,530 (2,300-11,820)	6,110 (2,570-12,170)	6,220 (2,300-12,170)	0.238
Hemoglobin, mean (SD) (g/dL)	13.73 (1.73)	13.40 (1.64)	13.49 (1.67)	0.223
Platelet, mean (SD) (×10^9^/L)	176.49 (61.99)	169.75 (79.57)	171.56 (75.15)	0.578
Creatinine, mean (SD) (mg/dL)	0.90 (0.40)	0.91 (0.37)	0.91 (0.38)	0.529
FIB-4 score^†^, median (range)	2.53 (0.47-13.32)	3.25 (0.67-18.65)	2.97 (0.47-18.65)	0.012^∗^
Low risk	11 (20.7)	15 (10.4)	26 (13.2)	
Indeterminate risk	17 (32.1)	45 (31.3)	62 (31.5)	
High risk	25 (47.2)	84 (58.3)	109 (55.3)	

VCTE: vibration-controlled transient elastography; dB: decibels; kPa: kilopascal; F: liver fibrosis stage; BMI: body mass index; HCV: hepatitis C virus; AST: aspartate aminotransferase; ALT: alanine aminotransferase; ALP: alkaline phosphatase; INR: international normalized ratio; FIB-4: fibrosis-4 index. ^†^Patients who scored <1.3 and >2.67 on FIB-4 were considered low and high risk for advanced fibrosis, respectively.

**Table 2:** Multivariable logistic regression analysis of factors associated with hepatic steatosis.

	Crude OR (95% CI)	*P* value	Adjusted OR (95% CI)	*P* value
BMI category				
Normal (<23)	1		1	
Overweight (23-24.9)	1.13 (0.33-3.81)	0.840	1.13 (0.34-3.80)	0.840
Obese (≥25)	8.41 (3.34-21.16)	<0.001^∗^	8.41 (3.34-21.16)	<0.001^∗^
Diabetes mellitus	3.37 (1.26-9.04)	0.016^∗^	3.37 (1.26-9.04)	0.016^∗^
AST (Ref = ≤1 × ULN)	0.99 (0.98-0.99)	0.018^∗^	0.99 (0.98-1.00)	0.200
ALP (Ref = ≤1 × ULN)	0.99 (0.98-0.99)	0.033^∗^	0.99 (0.98-1.01)	0.291
FIB-4 (Ref = <1.3)	0.87 (0.76-0.99)	0.036^∗^	0.96 (0.81-1.15)	0.684

BMI: body mass index; AST: aspartate aminotransferase; ALP: alkaline phosphatase; ULN: upper limit of normal; FIB-4: fibrosis-4 index; OR: odds ratio; 95% CI: 95% confidence interval.

## Data Availability

Data cannot be shared publicly because consent for publication of raw data was not obtained, and the dataset could, in theory, pose a threat to the confidentiality of the study participants. The dataset used and/or analyzed during this study are available on reasonable request from the ethics committee of Rajavithi Hospital (contact via e-mail: ec.rajavithihospital@gmail.com) for researchers who meet the criteria for access to confidential data.
